# Variation and Genetic Structure in *Platanus mexicana* (Platanaceae) along Riparian Altitudinal Gradient

**DOI:** 10.3390/ijms16012066

**Published:** 2015-01-19

**Authors:** Dulce M. Galván-Hernández, J. Armando Lozada-García, Norma Flores-Estévez, Jorge Galindo-González, S. Mario Vázquez-Torres

**Affiliations:** 1 Instituto de Biotecnología y Ecología Aplicada (INBIOTECA), Universidad Veracruzana, Av. de las Culturas Veracruzanas No. 101 Col. Emiliano Zapata, Xalapa 91090, Mexico; E-Mails: nflores@uv.mx (N.F.-E.); jorgegalin@gmail.com (J.G.-G.); 2 Facultad de Biología, Universidad Veracruzana, Zona Universitaria, Circuito Gonzalo Aguirre Beltrán s/n, Xalapa 91090, Mexico; 3 Centro de Investigaciones Tropicales, Universidad Veracruzana, Ex Hacienda Lucas Martín, Privada de Araucarias s/n, Col. Periodistas, Xalapa 91019, Mexico; E-Mail: savazquez@uv.mx

**Keywords:** altitude, gene flow, genetic drift, ISSR, sycamore

## Abstract

*Platanus mexicana* is a dominant arboreal species of riparian ecosystems. These ecosystems are associated with altitudinal gradients that can generate genetic differences in the species, especially in the extremes of the distribution. However, studies on the altitudinal effect on genetic variation to riparian species are scarce. In Mexico, the population of *P. mexicana* along the Colipa River (Veracruz State) grows below its reported minimum altitude range, possibly the lowest where this tree grows. This suggests that altitude might be an important factor in population genetics differentiation. We examined the genetic variation and population structuring at four sites with different altitudes (70, 200, 600 and 1700 m a.s.l.) using ten inter-simple sequence repeats (ISSR) markers. The highest value for Shannon index and Nei’s gene diversity was obtained at 1700 m a.s.l. (*H*e = 0.27, *N*e = 1.47, *I* = 0.42) and polymorphism reached the top value at the middle altitude (% *p* = 88.57). Analysis of molecular variance (AMOVA) and STRUCTURE analysis indicated intrapopulation genetic differentiation. The arithmetic average (UPGMA) dendrogram identified 70 m a.s.l. as the most genetically distant site. The genetic structuring resulted from limited gene flow and genetic drift. This is the first report of genetic variation in populations of *P. mexicana* in Mexico. This research highlights its importance as a dominant species, and its ecological and evolutionary implications in altitudinal gradients of riparian ecosystems.

## 1. Introduction

The sycamore, *Platanus mexicana*, is an arboreal species distributed on the slopes of the Gulf of Mexico, the Gulf of Tehuantepec and Guatemala. It is mainly found naturally near streams and river banks, where it is a dominant species. It is also found in cities, planted as an ornamental tree, because of its quick growth and resistance to air pollution [1]. Riparian ecosystems have important ecological functions such as functional connectivity, increased dispersal of plant species, mitigation of the processes of sedimentation in riverbeds, maintaining water quality, decomposition and nutrient cycling, and providing flood and erosion protection [2]. Riparian ecosystems are associated with altitudinal gradients that provide significant environmental variations [3]. Nevertheless, population genetic research relating to the riparian-altitudinal gradients are rare in the context of specific tree species, in particular for elucidating potential effects of altitude on patterns of genetic variation.

There are no studies of genetic variation in *P. mexicana* related to altitudinal gradients. The altitudinal genetic variation of riparian vegetation is affected by several factors, including ecological ones such as landscape characteristics (altitude, stream flow), landscape fragmentation, sexual reproduction, and flowering phenology [4,5]. Moreover, evolutionary processes such as bottlenecks, genetic drift or barrier to gene flow have a significant influence as they enhance genetic differentiation between populations [6]. As such, the effect of an altitude gradient on the genetic variation of a species depends on a group of factors that result in site variation, and can even lead to local adaptation and population differentiation [7].

Gene flow through pollen and seed dispersion is the most important factor in genetic exchange between populations [8]. In riparian species this flow can occur in two ways: bidirectional or unidirectional. The bidirectional movement can maintain homogeneous genetic variation in the gradient; this was observed in *Fraxinus mandshurica* [3] and *Euptelea pleiospermum* [5]. The unidirectional movement promotes the genetic differentiation by accumulation of genetic variation either upstream or downstream of the altitudinal gradient; this was observed in *Myricaria laxiflora* whose seed movement by hydrochory increased heterozygosity in the lower part of the gradient [9]. The evolutionary implications in populations are not yet defined, however, it is possible that genetic impoverishment in some extreme altitudinal gradients increases extinction risk. Additionally, if we add the effect of other factors such as bottleneck, this risk is enhanced [10].

The reduction in population size can cause random alterations in allele frequencies (genetic drift). The allele frequency can undergo large fluctuations in different generations in an unpredictable pattern and can result either in fixation or loss of alleles [8]. However, organisms can be affected by the processes of selection due to the different environmental conditions along gradients. This is mainly observed in populations located at the limits of the species’ distribution. Evidence of this was found in a work with a *Pinus hartwegii* population, in which the effect of selection was inferred from the number of low frequency alleles present in the subpopulations of the altitudinal gradient’s end [7,11].

The edges of distribution of a species may affect genetic variation in populations because in these places environmental conditions are often adverse or unusual for individual establishment [7]. In the case of *P. mexicana*, their populations are in a normal distribution range between 600 and 1800 m a.s.l., however, populations growing at altitudes of 160 to 2400 m a.s.l. have been reported [12]. In the northeastern region of the state of Veracruz, Mexico a population of *P. mexicana* along the Colipa River grows at altitudes as low as 70 m a.s.l. For this reason, the aim of this study is to analyze the genetic variation of *P. mexicana* and understand its ability to respond to different environmental conditions along an altitudinal gradient, especially in unusual conditions for the species. Since there are no records of this species growing at such a low altitude, the study also aims to understand the ecological and evolutionary factors that promote genetic differentiation. Inter-simple sequence repeats (ISSR) molecular markers were used because of their high variability [13] and the lack of *P. mexicana* genomic sequences. This is the first study that reports the effect of riparian altitudinal gradients on the variation and genetic differentiation of *P. mexicana*.

## 2. Results

### 2.1. Genetic Diversity

Ten selected ISSR primers generated a total of 105 bands. The size range of polymerase chain reaction (PCR) fragments was 180–1700 bp. The number of bands and the percentage of polymorphisms varied with each primer. Primer 857 produced the highest number of bands (15), of which 14 were polymorphic (Table 1).

**Table 1:** ISSR primers used to analyze genetic diversity on populations of *P. mexicana* at different altitudes.

Primer	Sequence	Size Range	No. of Bands	No. of Polymorphic Bands
818	(CA)_8_G	350–1000	12	9
824	(TC)_8_G	350–1500	9	8
827	(AC)_8_G	400–1400	9	8
829	(TG)_8_C	480–1700	7	4
835	(AG)_8_YC	180–1400	9	7
841	(GA)_8_YC	300–1200	9	7
845	(CT)_8_RG	250–1500	12	10
848	(CA)_8_RG	300–1500	10	9
850	(GT)_8_TYC	300–1500	13	11
857	(AC)_8_YG	180–1500	15	14

Analysis of the results showed significant differences in the genetic variation of the Colipa River populations of *P. mexicana* with respect to its distribution on the altitudinal gradient. At 1700 m a.s.l. Nei’s gene diversity (*H*e), effective number of allele per locus (*N*e) and Shannon index (*I*) exhibited the highest values compared to the other sites (*H*e = 0.27, *N*e = 1.47 and *I* = 0.42; Table 2). The lowest level of variability was observed at 70 m a.s.l. (*H*e = 0.22, *N*e = 1.37 and *I* = 0.35). There was a gradual decrease of these parameters as the altitude of the sampled populations decreased. Comparison between He, Ne and I among sites, revealed that both ends of the gradient display significantly different values (*H*e:*Z* = 3.13, *p* = 0.001; *N*e:*Z* = 2.91, *p* = 0.003; *I*:*Z* = 3.07, *p* = 0.002; Table 2). Although the Wilcoxon test was not significant for the percentage of polymorphic loci, it is worth noting that the highest value was recorded at the mid-altitude sites, 200 and 600 m a.s.l. (88.5%), and the lowest value was recorded at 70 m a.s.l. (81.9%). The values for the number of alleles per locus (A) were similar among sites, except between 70 and 200 m a.s.l. (Table 2).

**Table 2:** Parameters of genetic variation in *Platanus mexicana* on the Colipa River, Veracruz, Mexico.

Elevation (m a.s.l.)	*H*e	*% p*	*A*	*N*e	*I*
70	0.228 ^a^	81.905 ^a^	1.352 ^a^	1.378 ^a^	0.352 ^a^
200	0.257 ^a,b^	88.571 ^a^	1.543 ^b^	1.430 ^a,b^	0.396 ^a,b^
600	0.265 ^a,b^	88.571 ^a^	1.448 ^a,b^	1.449 ^a,b^	0.402 ^a,b^
1700	0.279 ^b^	87.619 ^a^	1.514 ^a,b^	1.475 ^b^	0.424 ^b^

*H*e = Nei’s gene diversity; % *p* = percent polymorphism; *A* = number of alleles per locus; *N*e = effective number of alleles per locus; *I* = Shannon Index; Wilcoxon’s test (*p* < 0.05); different letters indicate a significant difference between values.

### 2.2. Genetic Differentiation and Population Structure

**Figure 1:**
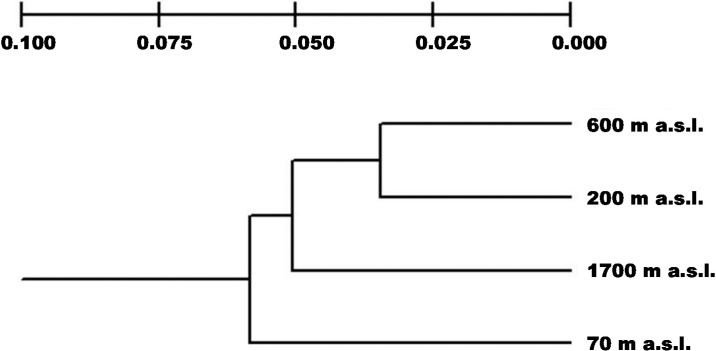
UPGMA diagram based on Nei’s genetic distances for *P. mexicana* on the Colipa River, Veracruz, Mexico.

**Figure 2:**
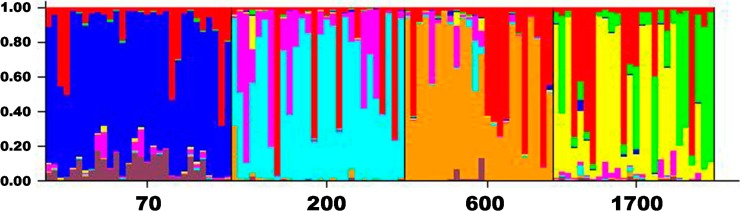
Population structure based on ISSR variation among sites at *K* = 8, implemented in STRUCTURE, v. 2.3, where each line represents the proportional assignment of an individual to the clusters, represented by the different colors.

The AMOVA analysis corroborated the genetic structure, the value of Φ_ST_ that represents the genetic differentiation among sites was 0.2 (*p* = 0.001); the main variation component (80%) was attributable within sites. Paired AMOVA showed the existing differentiation between sites mainly between 70 m a.s.l. and the others elevations (Table 3). A UPGMA dendrogram (Figure 1) based on Nei’s genetic distances exhibited three groups; one formed by the intermediate sites between 200 and 600 m a.s.l., (0.034), followed by the site at 1700 m a.s.l. (0.05) and the most distant being the site at the lowest altitude 70 m a.s.l., (0.058). The most likely number of clusters (*K*) in STRUCTURE was determined using the Δ*K* method. We assumed *K* = 8 as the best model that explains the genetic structure of *P. mexicana* population in the Colipa river (Figure 2) (See Supplementary Table S1) where well-defined altitude groups are observed. This suggests strong intra-population genetic differentiation. The Mantel test revealed that the pairwise Φ_ST_ are not correlated with geographic distances (*r* = 0.44, *Z* = 20.98, *p* = 0.251) and so the hypothesis of isolation due to distance was discarded.

**Table 3:** Paired AMOVA between sites on the Colipa River.

Sites (m a.s.l.)	Φ_ST_
70 & 200	0.238 *
70 & 600	0.216 *
70 & 1700	0.198 *
200 & 600	0.166 *
200 & 1700	0.176 *
600 & 1700	0.169 *

* Statistically significant values (*p* = 0.001).

### 2.3. Genetic Drift

The comparison of loci with allele frequencies lower than 0.05 (Wilcoxon *p* < 0.05) confirmed the effect of genetic drift at the lowest site, given that it had a significantly higher number of rare-fixed alleles relative to the sites at 200 m a.s.l. (*p* = 0.02) and 1700 m a.s.l. (*p* = 0.002, Table 4).

**Table 4:** Between-site comparison of allele frequency at 0.05.

Sites Compared (m a.s.l.)	Valid *N*	*Z*	*p* Level
70 & 200	54	2.292338	0.021887 *
70 & 600	54	1.357806	0.174526
70 & 1700	54	2.973587	0.002944 *
200 & 600	54	1.158860	0.246514
200 & 1700	54	0.909226	0.363231
600 & 1700	54	0.909226	0.363231

*Z*, standard normal distribution; *N*, sample number; * Statistically significant values for the Wilcoxon Matched Pairs Test, *p* < 0.05.

## 3. Discussion

### Genetic Diversity and Differentiation

Based on the obtained results genetic differences were observed in *P. mexicana* on the altitudinal gradient along the Colipa River. Values of I and He were similar to one of the altitude patterns described by Ohsawa and Ide [14] where the genetic variation was greater at higher altitudes. According to their results this pattern has been observed in herbaceous rather than tree species due to processes of adaptation to severe conditions, historical movements and mutation rates. However, there is a risk of assuming that in a small basin, the historical movements or mutation rates are the cause of genetic differentiation within the Colipa River.

Our results provide evidence of the influence of an altitudinal gradient on genetic differentiation in the populations of *P. mexicana*. Although the UPGMA separates the populations into three significantly different groups, STRUCTURE provides a more detailed analysis of intra-population genetic structure being observed in allelic groups corresponding to each altitudinal site. The fact that there is greater variation within sites in the Colipa River area could be the result of outcrossing with a completely random mate. This was observed in *Monimopetalum Chinense* and outcrossing woody species [15]. Population structure in *P. mexicana* could be attributable to a combination of bottlenecks, genetic drift and natural selection, mainly in the lower part of the gradient.

Paired AMOVA showed that sites differ from each other with the greater differentiation being between 70 m a.s.l. and the other sites. In contrast, the smallest differentiation occurs between mid-altitudes, this may be the result of gene flow between sites which is reflected in the UPGMA explaining why the middle sites are in the same group. This suggests that gene flow might play an important role on the genetic structure observed due to the non-homogeneous values of genetic diversity between altitudinal sites. In wind-pollinated species such as *P. mexicana*, gene flow is expected to be constant in the altitudinal gradient by the influence of strong winds. In some anemophilous species, the bidirectional movement of pollen along a river can cause homogeneity in genetic variation. This was observed in *Fraxinus mandshurica* [3], *Pinus oocarpa* [16], *Quercus serrata* [17], *Larix kaempferi* [18] and *Picea abies* [19], where gene flow was constant among their populations. The Colipa River has a south-northeast orientation, with the southern part at the highest elevation and the lowest part only a few kilometres from the coast where the river empties into the Gulf of Mexico. Uphill winds could favor the unidirectional movement of pollen and explain the higher degree of genetic diversity of *P. mexicana* at the top of the gradient. But even though the pollen of *P. mexicana* can travel up to 2750 m, the dispersal is limited to 600 m a.s.l. [20]; this has great influence on the genetic differentiation that the populations presented. Also, according to Premoli [4], if the flowering phenology varies along an elevation gradient, gene flow between low-elevation and high-elevation populations is unlikely to occur. This is an issue that has not been clarified in *P. mexicana*.

On the other hand, dispersal of seeds not only causes the movement of new genotypes, but also determines how these are distributed among different microsites and therefore might have a larger influence on the local genetic structure [21]. In riparian species this is favored by hydrochory, as is the case of *Myricaria laxiflora* whose seed movement heterozygosity increases towards the lower part of the gradient [9]. If such movement existed in *P. mexicana*, it would be expected that the values of genetic variation would be higher than those presented at lower altitudes, but this was not observed.

In riparian species, abiotic factors such as strong winds and recurring torrential floods, affect trees established in the lower part of a basin, this creates problems of colonization and retention [2] causing reductions in population size mainly in fragmented populations [22]. The reduction of genetic variation observed in *P. mexicana* at the lowest part of the gradient may be the result of this effect that could be considered as recurrent bottlenecks. For example, in *Picea glehnii* at lower elevations in the Furano region of central Hokkaido in Japan, Goto *et al.* [23] found that it was very likely that a bottleneck had been established in this population by isolation as a result of past demographic history. However, the methodology used in our research was unable to determine such events for populations of *P. mexicana* in the Colipa River.

In peripheral populations, reduced genetic variation may also be linked to selective pressures under harsh climatic conditions [24]. In populations near their distribution edge, natural selection is a force differentiating those areas from the rest of distribution and conferring an adaptive advantage to certain loci [7,25]. This could lead to the presence of low frequency alleles as an adaptation mechanism to unfavorable environments, as it was reported in *Pinus hartwegii* where the effect of selection was inferred from the number of low frequencies alleles present in a population in extreme conditions [11]. Presence of *P. mexicana* at 70 m a.s.l. suggests an expansion of its altitudinal distribution range on the Colipa River. The results of our research could serve as the basis for further studies about these Platanus populations.

## 4. Materials and Methods

### 4.1. Study Area

**Figure 3:**
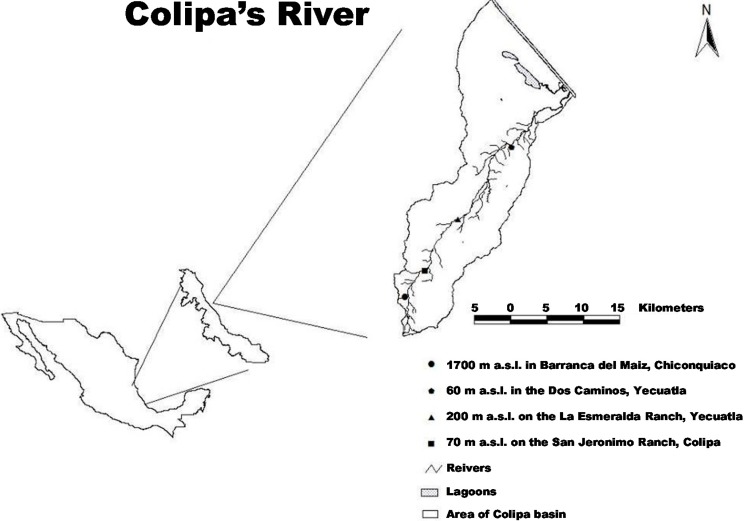
Location of four sampled sites of *P. mexicana* in the Colipa River.

The study area is located along the Colipa River in the northeastern part of the state of Veracruz, Mexico. Due to the length of the river (40.68 km) and the fact that its altitude ranges from sea level to 1700 m a.s.l., four sites were chosen along this gradient: at 1700 m a.s.l., in “Barranca del Maíz”, Chiconquiaco Municipality (19°47'28''N, 96°48'50''W) with a mean annual temperature of 20 °C; at 600 m a.s.l. in “Dos Caminos”, Yecuatla Municipality (19°49'26''N, 96°47'43''W) with a mean annual temperature of 22 °C; at 200 m a.s.l. on “Rancho La Esmeralda”, Yecuatla Municipality (19°53'15''N, 96°44'57''W) with a mean annual temperature of 24 °C; and the site at 70 m a.s.l. on “Rancho San Jerónimo” in the Colipa Municipality (19°58'36''N, 96°40'58''W) with a mean annual temperature of 26 °C (Figure 3).

### 4.2. Collection and Preservation of Plant Material

At each site, 30 trees were randomly selected and healthy mature leaves with no signs of physical damage were collected from them. The leaves were washed in the field in a soapy solution with 5% bleach for five minutes, after which they were rinsed with distilled water and cleaned with cotton batting dampened with 70% alcohol, rinsed again in distilled water and dried with paper towels. One hundred 1 cm^2^ squares of leaf tissue were cut per tree and stored in small plastic bags with an air-tight seal and silica gel inside to keep them dry until their analysis [26].

### 4.3. DNA Extraction and ISSR Amplification

DNA extraction was done using the CTAB method [27,28], modified as follows: 0.7 g of dry leaf tissue was ground in liquid nitrogen, and 1 mL of CTAB 2× extraction buffer (100 mM Tris–HCl pH 8.0, 1.4 M·NaCl, 20 mM EDTA, 2% hexadecyltrimethylammonium bromide “CTAB”, 0.5% PVP, and 0.2% β-mercaptoethanol) was added to each sample. DNA amplification was done following [29] modified as follows: decreasing the quantity of DNA and PCR buffer; increasing the concentration of MgCl_2_ and dNTPs and eliminating the use of formamide. Only the samples showing successful amplification for the ten primers were used for further analysis. The ISSR primers used were divided according to annealing temperature in group 1:818, 824, 827 and 829; and group 2:835, 841, 845, 848, 850 and 857. PCR reactions were performed using a total volume of 20 μL, using 10 ng DNA, 1× PCR buffer (NH_4_)SO_4_, 3 mM of MgCl_2_, 0.375 mM of dNTPs, 1 µM of each primer, 1 unit of Taq-DNA polymerase (Fermentas, Waltham, MA, USA) and double distilled water. The amplification parameters were: initial denaturation for 4 min at 94 °C; 35 cycles of 30 s at 94 °C, 45 s at the annealing temperature (50 °C for group 1, 53 °C for group 2), 90 s at 72 °C; followed by a final extension of 8 min at 72 °C. Amplification products were separated on a 1.5% agarose gel on 1× TBE buffer, stained with ethidium bromide and followed by photo documentation using Kodak ID 3.5.

### 4.4. Data Analysis

All the amplified bands were treated as dominant genetic markers. ISSR bands were scored as 1 (present) or 0 (absent) and this binary data was used to assemble a rectangular matrix. Allele frequencies *p* and *q* were inferred using the Lynch and Milligan adjustment for dominant markers, were recessive allele is determined by $q=\sqrt{\left(\frac{N0}{N0+N1}\right)}$ and dominant allele by p=1−q [30]. The percentage of polymorphic loci (% *p*), number of alleles per locus (A), effective number of alleles per locus (*N*e), Nei’s gene diversity (*H*e) and Shannon index (*I*) were calculated using the TFPGA v1.3 software [31]. To detect any differences in heterozygosity among elevations, Wilcoxon paired tests were run in STATISTICA, v8 [32].

To estimate the genetic differences among sites along the altitudinal gradient, an analysis of molecular variance (AMOVA) was run in GenAlEx, v. 6.41 software [33], this analysis produces variance components estimates that are similar to the F statistics of Wright, called Φ*_ST_*. In addition, paired AMOVAs were computed between sites to pairwise differentiation indices. To test the results of this analysis, genetic distances were inferred with the method of Nei and Lee [34], and similarity estimates were analyzed using Unweighted Pair Group Method with Arithmetic Averages (UPGMA) with TFPGA, v 1.3 software [31].

A further classification was made using STRUCTURE v. 2.3 software [35]. The RECESSIVEALLELE = 1 algorithm for dominant markers was used, where 0 = recessive allele for each locus. The number of inferred groups was evaluated at values of *K* ranging from 2 to 10, a burn-in length of 50,000 followed by 10 runs at each value of *K*. Estimates were obtained under the admixture model using the correlated allele frequencies and the LocPrior algorithm, which incorporates sampling location information and is appropriate for detecting weak population structure [36]. The most likely number of clusters (*K*) was determined using the Δ*K* method, as well as by examining the plateau of the Ln Pr(*X*/*K*) [37].

To test whether the genetic structure is a result of isolation because of distance, pairwise Φ_ST_ *versus* geographic distances were tested for correlation using Mantel’s test [38] run in TFPGA, v. 1.3. As another measure related to allele fixation from genetic drift, the quantity of fixed loci or those with allele frequencies lower than 0.05 between sites were tested using Wilcoxon’s test in STATISTICA, v. 8 [32].

## 5. Conclusions

Genetic differences were observed in *P. mexicana* along the altitudinal gradient of the Colipa River. It is necessary to restore the ecological importance of *P. mexicana* to riparian ecosystems; these ecosystems are in steady decline and sycamore is one of the most dominant species among them. In addition, there are problems of colonization and establishment of individuals especially in the lower part of the river. Individuals present in these areas must be resistant to adverse abiotic factors as previously mentioned, and adapted to support this type of environment. Thus the possibility of formation of new populations from a low number of trees in which genetic differentiation is low, favoring the presence of rare alleles and the scarcity of other common alleles as compared to the allelic frequency of the original population cannot be discarded. The results of this research highlight the importance of *P. mexicana* as a dominant species and its ecological and evolutionary implications as a key species of riparian ecosystems. The results presented in this report represent a basic knowledge of the genetic variability of this species in the Colipa River region.

## Supplementary Materials

Supplementary materials can be found at http://www.mdpi.com/1422-0067/16/01/2066/s1.
